# Different diagnostic criteria influence the determination of Vitamin D nutritional status in children: a cross-sectional study

**DOI:** 10.3389/fpubh.2025.1641065

**Published:** 2025-08-13

**Authors:** Qingling Zhu, Yueyuan Zhu, Jiajia Liu, Huiling Huang, Liuhong Huang, Weihua Lin

**Affiliations:** ^1^The School of Clinical Medicine, Fujian Medical University, Fuzhou, China; ^2^Department of Children Health Care, Quanzhou Women and Children’s Hospital, Quanzhou, China; ^3^Medical Clinic, Jilin University of the Arts, Changchun, China

**Keywords:** Vitamin D, 25(OH)D, children, criteria, cross-sectional study

## Abstract

**Objective:**

To assess Vitamin D status in children aged 0–6 years in Quanzhou, China, and compare the impact of diagnostic criteria on deficiency/sufficiency classification.

**Methods:**

This cross-sectional study enrolled 1,183 healthy children aged 0–6 years (January 2022–March 2023). Serum 25(OH)D levels were measured via ELISA, and anthropometric data were collected. Participants were stratified by sex, season, age, WLZ, BMI, and Vitamin A status to analyze Vitamin D variations. Diagnostic criteria impacts on classification were evaluated.

**Results:**

Mean serum 25(OH)D was 73.02 nmol/L (*IQR*: 58.48–89.09), with no sex-based differences (*P* > 0.05). Levels varied by season (highest in summer: 75.85 nmol/L; lowest in winter: 69.00), and age (infants > toddlers > preschoolers), all *P* < 0.05. Serum 25(OH)D were observed to decrease with increasing WLZ (Weight-for-Length Z-score) and BMI, though no statistically significant differences were found for either parameter (both *P* > 0.05) Using Criterion I, 2.46% were deficient, 9.97% insufficient, and 87.57% sufficient (*χ^2^* = 1589.053, *P* < 0.001). With Criterion II, rates shifted to 12.43, 40.74, and 46.83%, respectively (*χ^2^* = 239.271, *P* < 0.001). Classification discrepancies were significant across subgroups (sex, season, age, BMI; all *P_.adj_* <0.05), with poor inter-criteria agreement (*Kappa* = 0.071, *P* < 0.001). Age, season, BMI, and Vitamin A independently predicted sufficiency (*P* < 0.05).

**Conclusion:**

This study underscores two critical implications: (1) Vitamin D deficiency/sufficiency classifications are critically dependent on diagnostic criteria, necessitating region-specific guidelines and standardized threshold selection in practice and research. (2) Given the influence of latitude on Vitamin D synthesis, targeted interventions—particularly increased winter dosing for young children—should be tailored to age and seasonal variations.

## 1 Introduction

Vitamin D is an essential nutrient for infants and young children, as it plays crucial roles in growth, development, and overall health. The pivotal role of Vitamin D in maintaining skeletal health, especially during the critical developmental stages of preschool children, is supported by recent studies. In addition, studies have emphasized the multifaceted impact of Vitamin D on immune function, infections, allergies and cognitive development in children (1–3). Studies have shown that Vitamin D deficiency/insufficiency is a serious problem among healthy preschool children, highlighting the need for public health policies or interventions to ensure sufficient Vitamin D levels in this population (2, 4). The Centres for Disease Control and Prevention (CDC) also recommend Vitamin D supplements for children to help them obtain enough Vitamin D every day, with specific daily intake guidelines based on age (5). These recent studies and recommendations underscore the importance of maintaining sufficient Vitamin D levels in preschool children for the formation and mineralization of bones, and they advocate for the implementation of strategies to address Vitamin D deficiency in this population. Vitamin D insufficiency and deficiency are global problems that are becoming increasingly serious. Approximately 1 billion people worldwide are affected by Vitamin D deficiency, and approximately 50% of the global population has Vitamin D insufficiency (6, 7). The prevalence of Vitamin D deficiency was greater in adolescent, especially girls than in infant or toddler (8). Therefore, the international standardization of paediatric Vitamin D assessment criteria is essential for reliably monitoring global deficiency trends and guiding evidence-based interventions to prevent associated comorbidities and improve population health.

The assessment of optimal serum Vitamin D levels in preschool children is indeed complicated by the lack of uniformity in recommendations from health organizations worldwide. Furthermore, a review emphasized the need for new studies to assess the appropriateness of Vitamin D evaluation in selected cases, as universal screening of 25(OH)D serum levels may not be a feasible solution (9). The data on Vitamin D concentration and deficiency in healthy paediatric subjects are limited, indicating challenges in establishing uniform diagnostic criteria for Vitamin D status in children.

The discrepancies in the assessment of Vitamin D nutritional status due to variations in diagnostic criteria and recommendations from health organizations have raised concerns about the accuracy and consistency of reported prevalence rates of Vitamin D deficiency. These concerns are supported by various studies and clinical guidelines. The Endocrine Society’s Clinical Practice Guideline highlighted the lack of consensus on optimal serum 25(OH)D levels for different functional outcomes, emphasizing the need to re-evaluate the prevalence of Vitamin D deficiency, especially in children, based on the existing cut-off levels (10). A review article discussed the limitations of universal screening of 25(OH)D serum levels and the need for new studies to assess the appropriateness of Vitamin D evaluation in selected cases, considering the inconclusive data and diverse recommendations (9). Research on Vitamin D status in pre-schoolers in Montreal suggested that, despite variations in diagnostic criteria and recommendations, the Vitamin D status of children in this population was satisfactory, highlighting the complexity of interpreting prevalence rates of Vitamin D deficiency (9).

The prevalence of Vitamin D deficiency and insufficiency in children varies between studies and regions, mainly because of different reference values for Vitamin D dietary standards. This paper examines the nutritional status of Vitamin D in children less than 6 years of age in Quanzhou, China, according to various criteria and draws the attention of paediatricians worldwide to the criteria for assessing the nutritional status of Vitamin D in children.

## 2 Methods

### 2.1 Participants

The serum 25(OH)D levels of children aged 0–6 years who underwent routine physical examinations at the Department of Children’s Health Care, Quanzhou Women’s and Children’s Hospital, Quanzhou city, Fujian Province, from January 2022 to March 2023 were retrospectively analysed. Children with recent infections, abnormal liver and kidney function, endocrine disorders, genetic immunodeficiency diseases, and bone metabolism abnormalities that may affect 25(OH)D levels were excluded. This study was approved by the Ethics Committee of Quanzhou Maternal and Child Healthcare Hospital-Children’s Hospital (Approval No. 202013), and all guardians of the children signed an informed consent form.

The research project has been undergone procedures in Medical Research Registration and Record Filing Information System of National Public Health Security Information Platform, People’s Republic of China (No. MR-35-25-023179).

Assuming a target prevalence rate of *p* = 10%, a confidence level of 95% (*Z* = 1.96), and a margin of error *d* = 5%, the sample size is calculated as follows: *n* = (*Z*^2^·*p*·(1 − *p*))/*d*^2^ = 138.3. The initial sample size is thus 139. In consideration of a stratified sampling design with an effect size of 1.5 and a dropout rate of 20%, the estimated sample size is as follows: *N* = 139 × 1.5/(1 − 20%) = 260.6. Consequently, the final sample size should exceed 261 participants.

### 2.2 Determination of 25(OH)D

Fasting venous blood was collected from each subject and centrifuged at 3500 rpm for 15 min within 10 min, and a serum sample was extracted for the assay. The concentration of 25(OH)D was determined by an enzyme-linked immunosorbent assay (manufacturer Hefei Harmony Medical Technology Co., Ltd.). The inter- and intra-assay coefficients of variation were <10%.

### 2.3 Anthropometric methods

Anthropometric measurements (weight, length/height) were performed by trained staff following standardized protocols. Recumbent length was measured to the nearest 0.1 cm using an infantometer for children under 2 years of age. Standing height was measured to the nearest 0.1 cm using a stadiometer for children aged 2 years and older. Weight was measured to the nearest 0.1 kg using a calibrated electronic scale with participants wearing light clothing.

### 2.4 Grouping settings

All the subjects were divided into three groups according to their age: the infant group (≤1 years), the toddler group (>1 and ≤3 years) and the preschooler group (>3 and ≤6 years). The Vitamin D nutritional status of the children was assessed based on serum 25(OH)D concentrations, with classification into three distinct categories: Vitamin D deficient, Vitamin D insufficient and Vitamin D sufficient. In Criterion I (9), 25(OH)D < 30 nmol/L is deficient, ≥30 and <50 nmol/L is insufficient, and ≥50 nmol/L is sufficient. Whereas in Criterion II (8), these three values are <50 nmol/L, ≥50 and <75 nmol/L, and ≥75 nmol/L, respectively. Criterion I was selected as it represents the latest Chinese guidelines for paediatric Vitamin D assessment, whereas Criterion II reflects the other internationally used thresholds derived from global evidence. The children were divided into 4 groups according to the season: Spring Group (March, April and May), Summer Group (June, July and August), Autumn Group (September, October and November) and Winter Group (December, January and February). A serum Vitamin A concentration of ≥1.05 μmol/L was considered normal, and <1.05 μmol/L was considered insufficient (11).

Childhood obesity was diagnosed using criteria from the 2022 Expert Consensus on the Diagnosis, Evaluation and Management of Childhood Obesity in China (12). Consistent with standard paediatric anthropometric practice and this consensus, body mass index (BMI), calculated as weight (kg)/height (m^2^), was utilized only for participants aged ≥2 years. For children aged 2–5 years, overweight and obesity were defined using BMI reference cut-off points established in the *‘Body mass index growth curves for Chinese children and adolescents aged 0 to 18 years’* (13). For children aged 6–18 years, gender- and age-specific BMI reference cut-off points from the ‘*Screening for overweight and obesity in school-aged children and adolescents*’ were applied (14). For children aged <2 years, nutritional status was assessed using weight-for-length *Z*-scores (WLZ) according to WHO 2006 Child Growth Standards. WLZ was calculated relative to reference population means matched by age, sex, and length, with classification defined as: overweight (WLZ > +2 SD), obesity (WLZ > +3 SD), and normal (WLZ −2 SD to +2 SD) (15).

### 2.5 Statistical analysis

The statistical analysis was carried out using SPSS *26.0* software. Continuous variables, which followed a normal distribution, are expressed as the means ± SDs. Independent samples *t* tests were used for differences between two groups, whereas one-way ANOVA or analysis of covariance was used for differences among multiple groups. Continuous variables that did not follow a normal distribution were represented by *M* (*P_25_*–*P_75_*). Nonparametric tests for independent samples were used for differences between two groups, and nonparametric tests for *K* independent samples were used for differences among several groups. Classification variables are expressed as percentages, and differences between groups were tested using a cross-tabulated chi-square test. Linear regression analysis was used to analyse the effect of variables on a continuous variable; binary logistic regression analysis was used to analyse the effect of multiple variables on a categorical variable. Differences were statistically significant at *P* < 0.05. Comparisons between different diagnostic criteria were performed using the *Kappa* test.

## 3 Results

### 3.1 General information

A total of 1,183 subjects aged 0–6 years were recruited, including 704 boys and 479 girls. All the children were 2.00 (1.0–4.0) years old, with a height/length of 86.00 (74.00–99.20) cm and a weight of 11.4 (9.1–14.8) kg. Among the 560 infants under 2 years of age, recumbent length was 73.30 (69.10–79.00) cm, weight was 9.0 (8.00–10.00) kg, and WLZ was −0.13 (−0.89–0.61). WLZ were distributed as follows: 19 children (3.4%) had WLZ < −2, 531 children (94.8%) had WLZ ≥ −2 and ≤2, and 10 children (1.8%) had WLZ > 2. For children aged ≥2 years, standing height measured 91.20 (81.00–101.80) cm, weight was 12.90 (10.24–15.60) kg, and BMI was 15.36 (14.46–16.33). BMI were distributed as follows: 83 were overweight/obese (13.32%), 511 had normal-weight (82.02%), 29 were underweight (4.65%). 723 children (61.12%) had a Vitamin A concentration ≥1.05 μmol/L, and 460 children (38.88%) had a Vitamin A concentration <1.05 μmol/L (see Table 1).

**Table 1:** Common assessment criteria for Vitamin D nutritional status in children.

Category	Source details	Criterion I	Criterion II
Basic information	Source	Children’s Health Department of the Chinese Society of Preventive Medicine (11)	Amrein et al. (7) and Subspecialty Group of Endocrinologic, Hereditary and Metabolic Diseases, the Society of Pediatrics, Chinese Medical Association (30)
	Country	China	USA/USA
	Designated department	Children’s Health Department of the Chinese Society of Preventive Medicine	Endocrine Society in USA/Australia
	Year	2024	2020
Graduation	Deficient	<30 nmol/L	<50 nmol/L
	Insufficient	(30–50) nmol/L	(50–75) nmol/L
	Sufficient	≥50 nmol/L	≥75 nmol/L

### 3.2 Vitamin D status in children aged 0–6 years according to different diagnostic criteria

**Figure 1:**
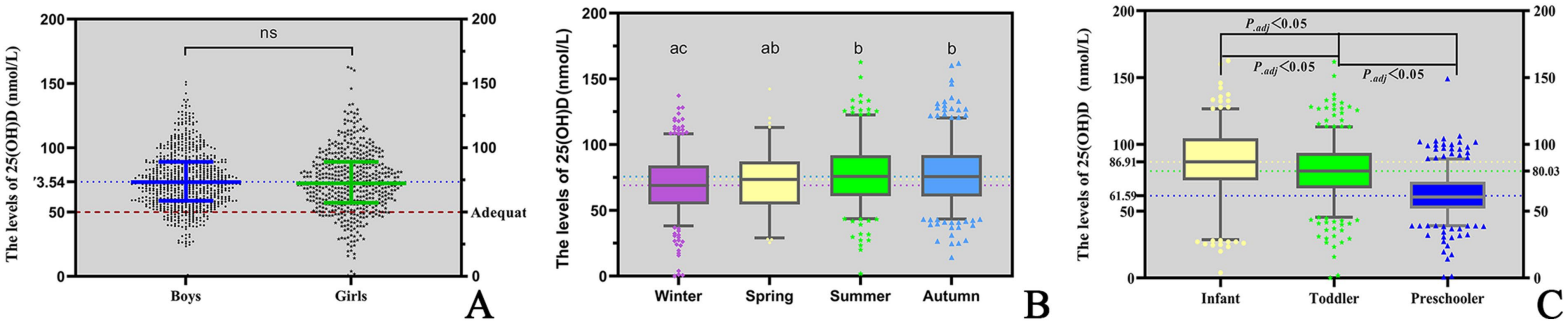
The levels of 25(OH)D. **(A)** Levels of 25(OH)D in children of different genders; **(B)** seasonal variations in serum 25(OH)D levels among children; **(C)** comparison of 25(OH)D levels across paediatric age groups. 1. “*ns*” means “not significant.” 2. “ABC” are the comparison results of seasonal differences in 25(OH)D levels by letter labelling method, *P._adj_* <0.05.

The serum 25(OH)D concentration was 73.02 (58.48–89.09) nmol/L in children aged 0–6 years in Quanzhou. There was no difference in the median Vitamin D concentration between the sexes (*P* = 0.416) (Table 2; Figure 1A).

**Table 2:** Comparison of serum 25(OH)D levels stratified by age, season, sex and weight-for-length/BMI.

Characteristic	Subgroup	*N*	25(OH)D nmol/L	*Z*	*P*
All children		1,183	73.02 (58.48–89.09)		
Sex	Male	704	73.47 (58.88–89.08)	−0.814	0.416
	Female	479	72.44 (57.32–89.11)		
Season	Spring	85	73.55 (54.46–87.22)	24.302	<0.001
	Summer	297	75.85 (60.77–91.94)		
	Autumn	405	75.71 (60.59–92.01)		
	Winter	396	69.00 (54.75–84.62)		
Age	Infant	252	86.91 (73.06–104.64)	278.800	<0.001
	Toddler	461	80.04 (67.44–93.71)		
	Preschooler	470	60.59 (51.94–72.07)		
Weight-for-length *Z* score	<−2	19	88.44 (77.66–106.99)	3.117	0.210
	≥−2, ≤2	531	84.88 (71.39–100.49)		
	>2	10	68.35 (57.39–94.19)		
BMI	Underweight	29	64.34 (52.63–82.06)	1.117	0.572
	Normal weight	511	64.34 (53.52–76.57)		
	Overweight/obese	83	62.43 (53.52–72.44)		

**Figure 2:**
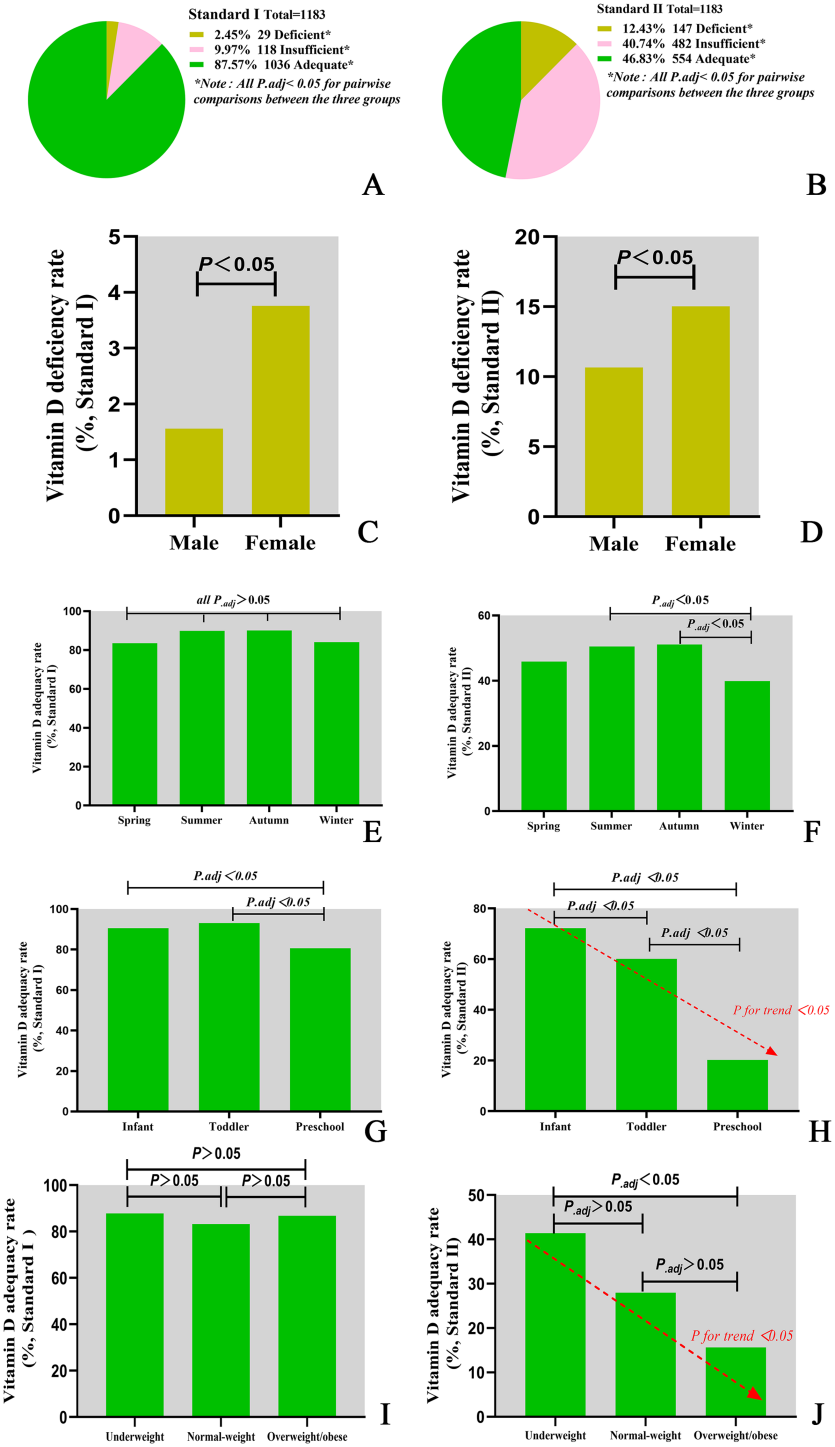
Vitamin D nutritional status classifications under different assessment criteria. **(A)** Vitamin D nutritional status classified by Criterion I. **(B)** Vitamin D nutritional status classified by Criterion II. **(C)** Vitamin D deficiency rate classified by Criterion I. **(D)** Vitamin D deficiency rate classified by Criterion II. **(E)** Vitamin D adequacy rate by season classified by Criterion I. **(F)** Vitamin D adequacy rate by season classified by Criterion II. **(G)** Vitamin D adequacy rate in different age groups classified by Criterion I. **(H)** Vitamin D adequacy rate in different age groups classified by Criterion II. **(I)** Vitamin D adequacy rate in different BMI groups classified by Criterion I. **(J)** Vitamin D adequacy rate in different BMI groups classified by Criterion II.

A significant seasonal difference in serum 25(OH)D levels was observed in children. The median Vitamin D concentration was highest in summer (75.85 nmol/L), followed by autumn (75.71 nmol/L), spring (73.55 nmol/L) and winter (69.00 nmol/L). The differences between two seasons are illustrated in Figure 1B. In addition, serum 25(OH)D levels significantly varied across different age groups. Infants presented the highest median Vitamin D concentration (86.91 nmol/L), followed by toddlers (80.04 nmol/L) and then preschoolers (60.59 nmol/L). The differences between each of the two age groups are illustrated in Figure 1C. Among infants under 2 years of age, serum 25(OH)D levels gradually declined with increasing WLZ, though this trend did not reach statistical significance (*p* ≥ 0.05). Similarly, in children aged ≥2 years, 25(OH)D levels were observed to decrease progressively with higher BMI (Table 2). Furthermore, the nutritional status of Vitamin D in children under the age of six in Quanzhou, China, was analysed according to different criteria. When grouped according to Criterion I, 29 (2.46%) were found to be deficient in Vitamin D, 118 (9.97%) were insufficient, and 1,036 (87.57%) were sufficient. The results were statistically significant. A significant difference was observed in the composition ratio between the groups (*χ^2^* = 1589.053, *P* < 0.001). Besides, both two-by-two comparisons between the groups were significantly different (all *P._adj_* <0.05), see Table 3 and Figure 2A. According to Criterion II, 147 (12.43%) patients were found to be deficient in Vitamin D, 482 (40.74%) were insufficient, and 554 (46.83%) were sufficient, with a statistically significant difference in the composition ratio between the groups (*χ^2^* = 239.271, *P* < 0.001). Furthermore, both two-by-two comparisons between the groups were significantly different (all *P._adj_* <0.05), see Table 3 and Figure 2B. No children with 25(OH)D levels above 250 nmol/L were identified, indicating that no children exhibited Vitamin D overdose or toxicity. There was a significant difference in the Vitamin D nutritional status between boys and girls. In accordance with Criterion I, there was a statistically significant difference in the prevalence of Vitamin D deficiency between boys and girls (*χ^2^* = 6.106, *P* < 0.05), the same result was also observed in criterion II (*χ^2^* = 668.065, *P* < 0.001). However, the remaining groups were not different (all *P_.adj_* >0.05), see Figures 2C,D. Regardless of whether Criterion I or II was met, there was no statistically significant difference in the sufficiency of Vitamin D among children of different sexes (all *P* > 0.05). However, there was a statistically significant difference in Vitamin D deficiency (all *P* < 0.05), with girls exhibiting significantly higher rates of deficiency than boys did.

**Table 3:** Comparison of Vitamin D nutritional status stratified by season and sex.

Subgroup	Characteristic	*N*	Vitamin D nutritional status (Criterion I/Criterion II)			*χ^2^* (Criterion I/Criterion II)	*P* (Criterion I /Criterion II)
			Deficient	Insufficient	Sufficient		
All children		1,183	29 (2.46%)/147 (12.43%)	118 (9.97%)/482 (40.74%)	1,036 (87.57%)/554 (46.83%)	1589.053/239.271	<0.001/<0.001
Sex	Male	704	11 (1.56%)/75 (10.65%)	64 (9.09%)/299 (42.47%)	629 (89.35%)/330 (46.88%)	7.589/5.591	0.022/0.059
	Female	479	18 (3.76%)/72 (15.03%)	54 (11.27%)/183 (38.21%)	407 (84.97%)/224(46.76%)		
Season	Spring	85	6 (7.10%)/14 (16.50%)	8 (9.40%)/32 (37.60%)	71 (83.50%)/39 (45.90%)	16.929/16.702	0.010/0.010
	Summer	297	6 (2.00%)/30 (10.10%)	24 (8.10%)/117 (39.40%)	267 (89.90%)/150 (50.50%)		
	Autumn	405	5 (1.20%)/40 (9.90%)	35 (8.60%)/158 (39.00%)	365 (90.10%)/207 (51.10%)		
	Winter	396	12 (3.00%)/63 (15.90%)	51 (12.9%)/175 (44.20%)	333 (84.10%)/158 (39.90%)		
Age	Infant	252	15 (6.00%)/24 (9.50%)	9 (3.60%)/46 (18.30%)	228 (90.50%)/182 (72.20%)	69.743/236.966	<0.001/<0.001
	Toddler	461	7 (1.50%)/32 (6.90%)	25 (5.40%)/152 (33.00%)	429 (93.10%)/277 (60.10%)		
	Preschooler	470	7 (1.50%)/91 (19.40%)	84 (17.90%)/284 (60.40%)	379 (80.60%)/95 (20.20%)		
Weight-for-length *Z* score	<−2	19	0 (0.00%)/0 (0.00%)	0 (0.00%)/3 (15.79%)	19 (100.00%)/16 (84.21%)	–^*^/–^*^	–^*^/–^*^
	≥−2, ≤2	531	19 (3.58%)/43 (8.10%)	24 (4.52%)/122 (22.98%)	488 (91.90%)/366 (68.93%)		
	>2	10	1 (10%)/2 (20.00%)	1 (10%)/4 (40.00%)	8 (80%)/4 (40.00%)		
BMI	Underweight	29	0 (0%)/5 (17.24%)	5 (17.24%)/12 (41.38%)	24 (87.76%)/12 (41.38%)	0.684/8.710	0.710/0.013
	Normal weight	511	8 (1.57%)/86 (16.83%)	78 (15.26%)/282 (55.19%)	425 (83.17%)/143 (27.98%)		
	Overweight/obese	83	1 (1.20%)/11 (13.25%)	10 (12.05%)/59 (71.08%)	72 (86.75%)/13 (15.66%)		

^*^The chi-square test could not be performed for underweight and overweight/obese children due to fewer than 5 cases in these subgroups.

There were notable variations in the nutritional status of Vitamin D across the seasons. According to Criterion I, there were statistically significant differences in the rates of Vitamin D deficiency, insufficiency and sufficiency among seasons (*χ^2^* = 16.929, *P* = 0.010). Furthermore, there were also statistically significant differences in the rates of sufficiency among seasons (*χ^2^* = 9.587, *P* = 0.022). However, no statistically significant difference was detected in the two-by-two comparisons between seasons (all *P_.adj_* >0.05), see Table 3 and Figure 2E. According to Criterion II, there was a statistically significant difference in the rates of Vitamin D deficiency, insufficiency, and sufficiency between seasons (*χ^2^* = 16.702, *P* = 0.010). Furthermore, there were statistically significant differences in the rates of Vitamin D sufficiency among seasons (*χ^2^* = 12.263, *P* = 0.007), with rates being higher in the summer and autumn than in the winter (all *P_.adj_*<0.05), see Table 3 and Figure 2F.

Moreover, Vitamin D nutritional status varied with age. According to Criterion I, there was a statistically significant difference in the deficiency, insufficiency and sufficiency of Vitamin D at different ages (*χ^2^* = 69.743, *P* < 0.001). Furthermore, the sufficiency of Vitamin D was significantly different across all age groups (*χ^2^* = 35.470, *P* < 0.001), see Table 3 and Figure 2G. The lowest sufficiency of Vitamin D was observed in preschool children, followed by infants, and the highest was observed in toddlers, see Figure 2G. According to Criterion II, there were statistically significant differences in the deficiency, insufficiency and sufficiency rates of Vitamin D at different ages (*χ^2^* = 236.966, *P* < 0.001). The sufficiency rates of Vitamin D were also found to vary significantly between age groups (*χ^2^* = 231.523, *P* < 0.001), with the highest rates observed in infants, followed by toddlers, and the lowest in preschoolers, see Table 3 and Figure 2H. Furthermore, there was a declining trend in the sufficiency of Vitamin D intake with increasing age (*P for trend* < 0.05), see Figure 2H.

Comparative analysis of Vitamin D sufficiency rates across WLZ categories could not be performed due to insufficient subgroup sample sizes. Nevertheless, the prevalence of Vitamin D sufficiency differed among children with varying BMIs. In accordance with Criterion I, there was no discernible difference in the Vitamin D sufficiency rate among children with varying BMIs (*P* > 0.05), see Figure 2I. However, according to Criterion II, there was a statistically significant difference in Vitamin D sufficiency among children with different BMIs (*P* < 0.001). The highest Vitamin D sufficiency was observed among children with low BMIs, followed by those with normal BMIs, and the lowest Vitamin D sufficiency was observed among those who were obese or overweight. Furthermore, Vitamin D sufficiency tended to decrease with increasing BMI. For further details, please refer to Table 3 and Figure 2J.

There was a statistically significant difference in the assessment of Vitamin D nutritional status between different diagnostic criteria (*Kappa value* = 0.071, *P* < 0.001).

### 3.3 Factors influencing Vitamin D sufficiency

**Figure 3:**
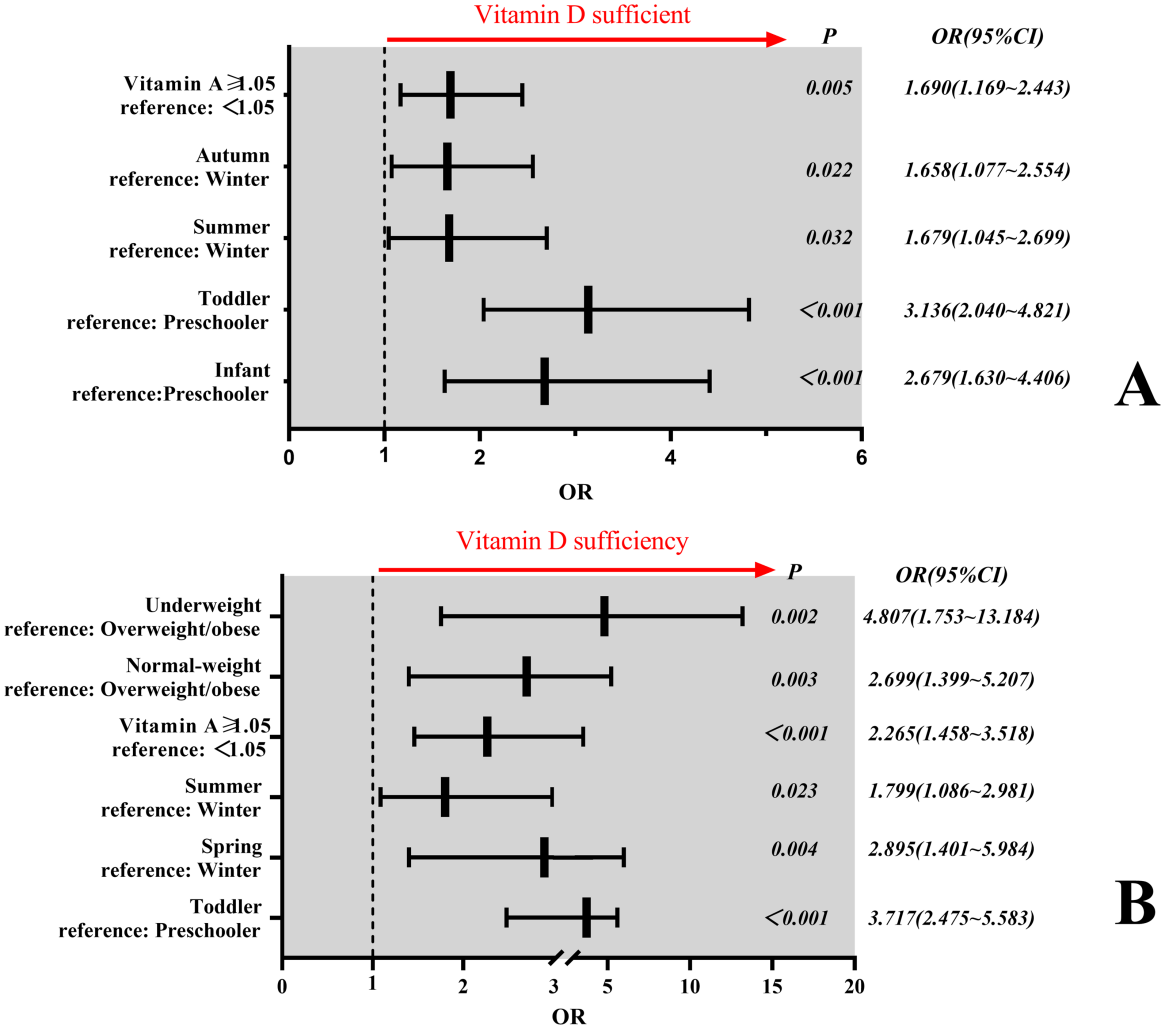
Factors influencing Vitamin D sufficiency. **(A)** Independent determinants of Vitamin D sufficiency under Criterion I. **(B)** Independent determinants of Vitamin D sufficiency under Criterion II. In panel **B**, only children aged ≥2 years (toddlers and preschoolers) were included in the regression analysis. Participants under 2 years of age were excluded due to the sample sizes of some subgroup <5 in univariate analysis, exceeding 20% of the total category and precluding meaningful statistical testing.

Binary regression analysis revealed that age, season, Vitamin A level and BMI independently influenced Vitamin D sufficiency. In Criterion I, age, season, and Vitamin A levels were found to be independent influencing factors for Vitamin D sufficiency (*P* < 0.001). Infants demonstrated 2.7-fold higher odds of Vitamin D sufficiency compared with preschool children [*OR* = 2.68; *95% CI* (1.63–4.41)], while toddlers exhibited 3.1-fold greater odds [*OR* = 3.14; *95% CI* (2.04–4.82)]. Children assessed during summer/autumn seasons had 1.7 times increased likelihood of Vitamin D sufficiency relative to winter evaluations [*OR* = 1.68; *95% CI* (1.05–2.70), and *OR* = 1.66; *95% CI* (1.08–2.55)]. Similarly, subjects with Vitamin A levels ≥1.05 μmol/L showed 1.7-fold higher odds of Vitamin D sufficiency versus those <1.05 μmol/L [*OR* = 1.70; *95% CI* (1.17–2.44)] (Figure 3A). In Criterion II, age, seasons, Vitamin A level, and BMI were found to be independent influencing factors for Vitamin D sufficiency (*P* < 0.05). Toddlers demonstrated 3.7-fold higher odds of Vitamin D sufficiency compared with preschool children [*OR* = 3.72; *95% CI* (2.48–5.58)]. Children assessed during spring had 2.9 times increased likelihood of Vitamin D sufficiency relative to winter evaluations [*OR* = 2.90; *95% CI* (1.40–5.98)], while children assessed during summer had 1.8 times increased likelihood of Vitamin D sufficiency relative to winter evaluations [*OR* = 1.80; *95% CI* (1.09–2.98)]. In a similar manner, subjects with Vitamin A levels ≥1.05 μmol/L showed 2.3-fold higher odds of Vitamin D sufficiency versus those <1.05 μmol/L [*OR* = 2.27; *95% CI* (1.46–3.52)]. Children with normal weight were 2.7 times more likely to be vitamin-D-sufficient than children with overweight/obesity [*OR* = 2.70; *95% CI* (1.40–5.21)]. Similarly, children with underweight were 4.8 times more likely to be vitamin-D-sufficient than children with overweight/obesity [*OR* = 4.81; *95% CI* (1.75–13.18)] (Figure 3B).

## 4 Discussion

In this study, we retrospectively analysed the levels of 25(OH)D in children aged 0–6 years in Quanzhou City over the past year. The level of 25(OH)D was found to be considerably higher than the threshold for Criterion I Vitamin D sufficiency (≥50 nmol/L) but slightly lower than the level for Criterion II Vitamin D sufficiency (≥75 nmol/L). With respect to Criterion I, the prevalence of Vitamin D deficiency was 2.46%, while the Vitamin D sufficiency rate was as high as 87.57%. In contrast, for Criterion II, the prevalence of Vitamin D deficiency increased to 12.43%, whereas the Vitamin D sufficiency rate decreased to 46.83%. There were no significant differences in 25(OH)D levels among children of different sexes. However, there were differences in Vitamin D nutritional status, with varying rates of deficiency, insufficiency, and sufficiency according to Criterion I. Most guidelines and reviews concur that a 25(OH)D level less than 25 or 30 nmol/L is detrimental to human health (16, 17). This criterion is consistent with Criterion I of our study, which yields a significantly lower prevalence of Vitamin D deficiency than the global prevalence (15.7%) (18).

Children’s 25(OH)D levels varied among seasons, with the highest being in summer and the lowest being in winter. In Criterion I, the 25(OH)D levels of children were sufficient in both summer and winter. However, in Criterion II, the 25(OH)D levels of children were only marginally sufficient, even during the summer months when there was a high level of sunlight. The prevalence of Vitamin D deficiency also varied seasonally, and this variation was significant for both Criteria I and II. The highest rates were observed in spring, at 7.1 and 16.5% for Criteria I and II, respectively, and lowest rates of Vitamin D deficiency were observed in autumn, at 1.2 and 9.9% for Criteria I and II, respectively. Vitamin D sufficiency was highest in autumn, with 90.1 and 51.1% for Criteria I and II, respectively; sufficiency was lowest in spring for Criterion I, with 83.5%, and in winter for Criterion II, with 39.9%. The different diagnostic criteria resulted in significant differences in sufficiency rates among seasons. The 25(OH)D levels of the children in this study were generally lower in the spring and winter than in the other two seasons, which is consistent with previous reports (19). Owing to our location at a lower latitude, specifically between 24°30′ and 25°56′, sunlight exposure is an effective method of promoting Vitamin D activation, even during the winter and spring months. This results in a prevalence of 25(OH)D levels <30 nmol/L, which represented only 2.46% of the population.

Latitudinal gradients significantly influence Vitamin D status, as evidenced by comparative analyses with other Chinese cohorts. In Suzhou (31.3°N, mid-latitude) (20), population-based data revealed a 42.1% prevalence of Vitamin D deficiency (25(OH)D < 50 nmol/L) among children aged 0–17 years—3.4-fold higher than our cohort (12.43% under Criterion II). Strikingly, Wuhan (30.6°N, similar latitude to Suzhou) (21) reported substantially higher sufficiency rates in preschoolers (72.7% *vs.* our 20.2% under Criterion II), despite its higher latitude than Quanzhou (24°–26°N). These contradictions highlight that latitude alone cannot explain geographic variability. Firstly, the 3.4-fold difference in deficiency levels observed between Suzhou and Quanzhou (42.1% *vs.* 12.43%) is consistent with the anticipated UVB attenuation at higher latitudes, thereby substantiating the pivotal role of latitude in modulating Vitamin D synthesis. Secondly, Despite comparable latitudes (~31°N) in Wuhan and Suzhou, Wuhan’s preschoolers showed 3.6 × higher sufficiency (72.7% *vs.* 20.2%)—suggesting dominant non-latitudinal factors (e.g., dietary practices, supplementation policies). Paradoxically, compared with Hangzhou (30.3°N) (4), Wuxi (31.57°N) (22) had higher deficiency (16.1% vs. 11.4% for <50 nmol/L) but also higher sufficiency (45.1% *vs.* 30.2% for ≥75 nmol/L), indicating complex threshold-dependent effects. Thus, while Quanzhou’s low latitude contributes to better Vitamin D status, regional heterogeneities in: Dietary patterns (e.g., seafood consumption in coastal Quanzhou), Public health interventions (e.g., Vitamin D fortification policies), Caregiver awareness (e.g., supplementation adherence), may override latitudinal advantages. This underscores the need for region-specific Vitamin D supplementation recommendations even within similar climatic zones.

Our data demonstrated an inverse relationship between BMI and vitamin D status. Although the trend of decreasing serum 25(OH)D levels with higher BMI did not reach statistical significance (*P* > 0.05; Table 2), diagnostic thresholds critically influenced sufficiency classifications. Under Criterion I, no significant difference in sufficiency rates was observed across BMI categories (*P* > 0.05; Figure 2I). However, Criterion II revealed a pronounced gradient: sufficiency rates declined progressively from 41.4% in underweight children to 28.0% in normal-weight and 15.7% in overweight/obese children (*χ^2^* = 8.71, *P* = 0.013; Table 3; Figure 2J). This threshold-dependent effect was quantified in regression analysis (Figure 3B): overweight/obese children had 4.8-fold lower odds of sufficiency (*95% CI:* 1.75–13.18) compared to underweight peers. Our findings align with Chen et al. (4), confirming that obese children exhibit the lowest absolute 25(OH)D levels (median: 62.43 nmol/L; Table 2) and lowest sufficiency rates. This likely reflects adipose sequestration of vitamin D, reducing bioavailability in higher-BMI individuals (23).

A meta-analysis (15) revealed that Vitamin D deficiency is more prevalent in women than in men, whereas only 2.2% of individuals <18 years old were affected. The Vitamin D nutritional status of boys and girls in this study was comparable, which is supported by other studies (4, 8, 9, 19). Therefore, the quantity of Vitamin D supplementation should not be solely determined by age but also by sex.

The observed poor agreement between diagnostic criteria (*Kappa* = 0.071) underscores a fundamental clinical dilemma: the two sets of cutoffs are not interchangeable and reflect divergent philosophies in defining Vitamin D adequacy. Criterion I (≥50 nmol/L) aligns with the Institute of Medicine (IOM) threshold emphasizing skeletal health outcomes, while Criterion II (≥75 nmol/L) adopts the Endocrine Society’s position targeting extraskeletal benefits (e.g., immune modulation) (10, 16). This discrepancy is not merely statistical but carries significant clinical implications: (1) For public health surveillance, Criterion I suggests a reassuringly low deficiency rate (2.46%), potentially reducing urgency for population-level interventions. (2) For individual risk stratification, Criterion II flags 12.43% as deficient and 40.74% as insufficient—highlighting potential extraskeletal risks in subgroups like preschoolers (20.2% sufficiency) and obese children (20.2% sufficiency). The disagreement primarily stems from arbitrary cutoff differences rather than biological variability. Our data show median 25(OH)D levels (73.02 nmol/L) lie precisely within the contested 50–75 nmol/L range. Consensus guidelines acknowledge this ambiguity: (1) The 2024 Endocrine Society Guideline maintains ≥75 nmol/L as optimal for “multiple health outcomes” but concedes ≥50 nmol/L suffices for bone health (10). (2) The IOM considers ≥50 nmol/L adequate for 97.5% of the population (16), but its applicability to children—especially for non-skeletal outcomes—remains debated. We recommend context-dependent standard selection: (1) Clinical practice: Prefer Criterion II for high-risk subgroups (obese children, preschoolers) to capture extraskeletal risks, as their sufficiency rates plummet to 20.2% under stricter thresholds. (2) Population surveillance: Criterion I may suffice for monitoring skeletal health trends, avoiding overestimation of deficiency in low-latitude regions like Quanzhou. Ultimately, harmonizing criteria requires pediatric-specific outcomes research linking 25(OH)D levels to functional endpoints (e.g., fracture risk, infection rates). Until then, explicit reporting of applied cutoffs is essential.

This study also sought to identify the factors influencing the sufficiency of 25(OH)D and Vitamin D in children. The age, season, Vitamin A levels and BMI of children in this region were found to significantly influence 25(OH)D levels, with these factors also being identified as independent influences on Vitamin D sufficiency. The observed association between Vitamin A insufficiency and reduced Vitamin D sufficiency (*OR* = 0.525–0.507, *P* < 0.005) warrants mechanistic exploration. We hypothesize three potential pathways. Firstly, dietary Co-Dependency. Vitamin A and D share common dietary sources (e.g., fortified dairy, fish liver oil, eggs). Children with Vitamin A insufficiency may concurrently lack Vitamin D-rich foods, explaining the correlated deficiency (24). Secondly, absorption Synergy. Both vitamins are fat-soluble and require biliary micelles for intestinal uptake. Vitamin A deficiency may impair fat absorption, reducing Vitamin D bioavailability—a phenomenon observed in celiac disease and cystic fibrosis (25). Thirdly, Molecular Crosstalk. Retinoic acid (Vitamin A metabolite) and 1,25(OH)D (Vitamin D metabolite) heterodimerize with retinoid X receptor (RXR) to regulate gene expression (26). Suboptimal Vitamin A levels may attenuate Vitamin D-dependent transcriptional activity, functionally mimicking insufficiency. While our study cannot establish causality, these hypotheses align with experimental evidence. First, Vitamin A-deficient rats show 30% lower 25(OH)D levels despite adequate D intake (27). Second, combined A supplementation improves bone mineral density more than either alone in deficient children (28, 29). Future studies should measure retinol-binding protein (RBP) and dietary logs to disentangle these mechanisms.

The application of disparate diagnostic criteria has led to considerable discrepancies in the fractional effects of age, season, Vitamin A levels and BMI on the level of 25(OH)D and Vitamin D sufficiency in children. Our analysis of two divergent criteria (Chinese *vs.* global) reveals that Vitamin D deficiency rates in Quanzhou children vary up to 5-fold depending on the applied standard. This discrepancy necessitates urgent policy attention: while regionally adapted criteria (Criterion I) account for local sunlight exposure, they may underestimate deficiency in high-risk subgroups (e.g., preschoolers, obese children) when compared to globally validated thresholds (Criterion II). Standardizing criteria across populations would improve surveillance accuracy and guide resource allocation for supplementation programs.

Despite geographic constraints, our core findings on diagnostic criteria discordance and demographic risk factors (e.g., 5.5× higher deficiency odds in obese children) align with global studies (7, 15, 20). This suggests that while absolute Vitamin D levels are region-dependent, the clinical imperative to standardize thresholds and target high-risk subgroups transcends local contexts.

## 5 Limitations

This study is inevitably subject to certain limitations. First, the cross-sectional design precludes causal inferences between variables. For instance, the observed inverse association between BMI and vitamin D levels could reflect reverse causation (e.g., obesity reducing vitamin D bioavailability) or residual confounding by unmeasured lifestyle factors. Second, while COVID-19 restrictions during the sampling period (January 2022–March 2023) likely limited children’s outdoor activities and sun exposure, we were unable to quantify the magnitude of this impact (e.g., reduced daily UV exposure hours) due to lack of behavioural data. Future longitudinal studies tracking vitamin D trends in the post-pandemic era are needed to clarify these dynamics. Third, potential residual confounding from unmeasured variables—such as precise dietary vitamin D intake, sunscreen use frequency, or genetic factors—may influence the observed associations. Finally, regional characteristics including Quanzhou’s latitude (24°–26°N), dietary patterns, and COVID-related restrictions may limit generalizability to populations with differing environmental or cultural contexts.

## 6 Conclusion

This study elucidates Vitamin D status in Quanzhou pre-schoolers, highlighting two critical implications: (1) Diagnostic criteria critically influence deficiency/sufficiency determinations, necessitating standardized threshold selection in clinical practice and research. (2) Given the established influence of latitude on Vitamin D synthesis, we recommend developing region-specific supplementation guidelines. Additionally, targeted interventions should be tailored to age and seasonal variations, such as increased winter dosing for young children.

## Data Availability

The raw data supporting the conclusions of this article will be made available by the authors, without undue reservation.
